# 21 novel pathogenic variants identified in a cohort of 77 Chinese families with osteogenesis imperfecta

**DOI:** 10.3389/fgene.2026.1825533

**Published:** 2026-04-29

**Authors:** Binshan Zhao, Chaoqun Zheng, Zhe Liu, Siji Zhou, Siyuan Tao, Xiuzhi Ren, Yaping Liu, Xiuli Zhao

**Affiliations:** 1 Center for Rare Diseases, State Key Laboratory of Complex, Severe, and Rare Diseases, Peking Union Medical College Hospital, Peking Union Medical College, Chinese Academy of Medical Sciences, Beijing, China; 2 Department of Medical Genetics, State Key Laboratory for Complex, Severe, and Rare Diseases, Institute of Basic Medical Sciences, Chinese Academy of Medical Sciences, School of Basic Medicine, Peking Union Medical College, Beijing, China; 3 Key Laboratory in Science and Technology Development Project of Suzhou (CN), Pediatric Orthopedics, Children’s Hospital of Soochow University, Suzhou, China

**Keywords:** Chinese cohort, genotype-phenotype correlation, osteogenesis imperfecta, phenotype, variant spectrum

## Abstract

**Objective:**

Osteogenesis imperfecta (OI) is a group of connective tissue disorders with significantly clinical and genetic heterogeneity, which is characterized by low bone mineral density, recurrent fractures and skeletal deformities. This study aimed to conduct clinical and genetic analyses in a Chinese OI cohort to expand the spectrum of pathogenic variants and provide evidence for precise genetic counseling and prenatal genetic diagnosis.

**Methods:**

A total of 77 Chinese families with clinically suspected OI were enrolled in this study. Clinical assessments at enrollment included physical examinations, X-ray imaging, and bone mineral density testing. Whole exome sequencing (WES) combined with Sanger sequencing was used to detect candidate pathogenic variants. Variant pathogenicity was evaluated via bioinformatics analysis and familial co-segregation analysis. In this OI cohort, the spectra of pathogenic variants, clinical phenotypes, and genotype-phenotype correlations were analyzed.

**Results:**

A 100% detection rate for pathogenic variants was achieved in the 77 families, with 79 variants identified in total. Among the 79 variants, 21 (26.6%) were novel variants founded across six OI-associated genes. Interestingly, apart from the correlation between different pathogenic genes and clinical phenotypes, we also discovered that the severity and phenotype of patients associated with the location of pathogenic variants within the type I collagen domain, exhibiting an aggravating trend from the amino terminus to the carboxyl terminus.

**Conclusion:**

Based on previous studies of large OI cohorts, we expanded the spectrum of pathogenic variants by identifying 21 novel ones. Meanwhile, we discovered that the location of pathogenic variants, particularly missense variants, in type I procollagen is correlated with the clinical manifestations and severity of patients. These findings will provide important evidence for the precise diagnosis and genetic counseling of the disease.

## 1 Introduction

Osteogenesis imperfecta (OI) is a group of rare, genetically heterogeneous connective tissue disorders characterized by reduced bone mineral density, recurrent fractures, short stature, and limb deformities, as well as a range of extra-skeletal manifestations including blue sclerae, dentinogenesis imperfecta (DI), hearing loss, ligamentous laxity, cardiac valve abnormalities, and pulmonary function impairment (Stoll et al., 1989; Sillence et al., 1979). With an estimated incidence of 1 in 10,000 to 20,000 live births (Forlino and Marini, 2016), OI affects individuals across all ethnic groups equally, and impose severe physical, psychological, and socioeconomic burdens on patients and their families (Rork et al., 2023).

The traditional classification of OI, proposed by Sillence, Senn, and Danks (1979), categorizes affected individuals into types I–IV based on clinical severity (Sillence et al., 1979). With advances in the understanding of OI pathogenesis, types V–XXIII have been defined based on both genetic and clinical characteristics (Forlino and Marini, 2016; Rauch and Glorieux, 2004).

Approximately 80%–90% of OI cases exhibit autosomal dominant inheritance and are caused by variants in *COL1A1*/*COL1A2*, encoding type I procollagens pro α1(I) and pro α2(I), respectively. The pathogenic variants in α1 and α2 chains of type I procollagen [proα1(I) and pro α2(I)] disrupt synthesis and processing of type I collagen (Rauch and Glorieux, 2004). In recent years, an increasing number of variants in other collagen-related genes including at least 20 non-type I collagen genes (e.g., *IFITM5*, *SERPINF1*, *WNT1*), have been reported to contribute to OI development as well. These genes affect processes such as procollagen post-translational modification, helical folding, osteoblast differentiation, and bone matrix mineralization (Jovanovic et al., 2022; Marini et al., 2014).

Since 2012, our team has been engaged in genetic and clinical research on Chinese OI cohorts, and has identified more than 200 novel pathogenic variants. Our previous work based on several Chinese OI cohorts has revealed significant differences between Chinese and other ethnic populations in major OI-causing genes, genotype-phenotype correlation, and the frequency of hotspot variants. However, many OI patients still carry unidentified pathogenic variants, and the genetic and clinical spectra of Chinese OI cohorts remains need to be further clarified (Li et al., 2019; Zhou et al., 2025).

In this study, we enrolled 77 families with OI, and performed clinical and genetic analyses to update and expand the clinical and genetic spectra of Chinese OI population, thereby providing solid evidence for the precise diagnosis and genetic counseling of the disease.

## 2 Materials and methods

### 2.1 Approval and informed consent

This study was approved by the Institutional Review Board of the Institute of Basic Medical Sciences, Chinese Academy of Medical Sciences, Beijing, China (Approval No. 015-2015), on 11 March 2015. Written informed consent was obtained from all adult participants, as well as from the legal guardians of underage participants.

### 2.2 Study participants and clinical evaluation

A total 77 Chinese families suspected of having OI were recruited from 2022 to 2025. The inclusion criteria for patients comprised a history of recurrent fractures, or obvious limb deformities, or vertebral compression fractures, with all patients exhibiting low bone mineral density. Metabolic disorders, such as hyperthyroidism or hypophosphatemic rickets disease, were excluded from the cohort by serum biochemical testing. Clinical data, medical histories, and peripheral blood or tissue samples were collected from the patients and their available family members.

Patients were divided into five clinical subtypes: Types I–IV were assigned according to the traditional Sillence classification (Sillence et al., 1979). Specifically, type I was represented the mildest form, characterized by blue sclerae and minimal fractures; type II was more severe, often leading to early death; type III was the most severe subtype among postnatal survivors; and type IV showed intermediate severity between Type I and Type III. Type V was defined by calcification of the interosseous membrane and growth of a hyperplastic callus formation at fracture sites (Glorieux et al., 2000).

### 2.3 Nucleic acid isolation

Genomic DNA (gDNA) was extracted from peripheral blood using the conventional proteinase K-phenol-chloroform method and quantified using NanoPhotometer N60 (Implen, Munich, Germany) for subsequent genetic analysis.

### 2.4 Whole exome sequencing (WES) and bioinformatics analysis

gDNA (1–3 µg) was randomly fragmented, end-repaired, and phosphorylated, followed by ligation with paired-end adaptors to construct sequencing libraries. After library size validation, high-throughput sequencing was performed on a HiSeq 4000 System (Illumina, Inc., San Diego, CA, United States). Raw sequencing image files were processed for base calling and quality filtering to generate high-quality clean reads. Clean reads were aligned to the human genome reference (hg19, GRCh37). Samtools (v1.19.2) was used for file sorting, and Picard (v2.25, MarkDuplicates) was used for duplicate marking. Variant calling was used to identify single-nucleotide polymorphisms (SNPs) and insertions/deletions (InDels), and all variants were annotated using multiple databases Synonymous single-nucleotide variants (SNVs) and variants with minor allele frequency (MAF) > 1% in the 1000 Genomes Project (1KGP) database were excluded for downstream analysis.

The Human Gene Mutation Database (HGMD, https://www.hgmd.cf.ac.uk/ac/ (Professional April 2024)), ClinVar (http://www.clinvar.com/ (accessed on 15 May 2019)), International Genome Sample Resource (IGSR, http://www.internationalgenome.org/(accessed on 22 September 2019)), Single Nucleotide Polymorphism Database (dbSNP, https://www.ncbi.nlm.nih.gov/snp (accessed on 29 February 2020)), and Genome Aggregation Database (gnomAD, http://www.gnomad-sg.org/ (accessed on 10 July 2020)) were used to assess the novelty of identified variants. For previously reported variants, pathogenicity was determined according to published database annotations. The pathogenicity of novel variants was assessed using *in silico* prediction tools and conservation analysis.

### 2.5 PCR and sanger sequencing

Candidate pathogenic variants identified by WES were validated via polymerase chain reaction (PCR) combined with Sanger DNA sequencing. Reference sequences of OI-related genes, including *COL1A1* (NM_000088.3)*, COL1A2* (NM_000089.3)*, IFITM5* ((NM_001025295.2)*, SERPINF1* ((NM_002615.5)*, TMEM38B* (NM_018112.3)*, FKBP10* (NM_021939.3)*, WNT1* (NM_005430.3), were retrieved from the University of California, Santa Cruz Genome Browser database (UCSC, http://genome.ucsc.edu/). Primers targeting variant regions were designed via Primer3 (http://primer3.ut.ee/) and verified by the UCSC BLAT tool.

gDNA amplification was performed using LA Taq polymerase with GC Buffer (TaKaRa Bio, Dalian, China) under the following thermal cycling conditions: initial denaturation at 95 °C for 3 mins; 35 cycles of denaturation at 94 °C for 30 s, annealing at 58 °C for 40 s, extension at 72 °C for 40 s; and a final extension at 72 °C for 8 min. Amplicons were sequenced on ABI 3730xl (Thermo Fisher Scientific, Waltham, MA, United States), and sequencing data were analyzed through CodonCode Aligner v6.0.2. The candidate pathogenic variants were further confirmed in the probands and their available family members.

### 2.6 Statistical analysis

Statistical analyses of phenotypic data were performed exclusively in the probands. The results were presented as mean ± standard deviation for normal distribution (such as height or age) and as percentages for binomial distribution (such as DI or blue sclerae). Differences between two or more groups were analyzed using the independent‐sample Student’s t-test, while differences among three or more groups were evaluated using one‐way analysis of variance (ANOVA). Welch’s t-test or Brown–Forsythe test was adopted as appropriate when Levene’s test indicated heteroscedasticity in the Student's t-test or ANOVA, respectively. Pearson’s chi‐squared test or Fisher’s exact test was used in the analysis of contingency tables, if appropriate. p values < 0.05 were considered statistically significant. All statistical analyses were conducted using SPSS Statistics (IBM, New York, NY).

## 3 Results

### 3.1 Clinical characteristics

**FIGURE 1:**
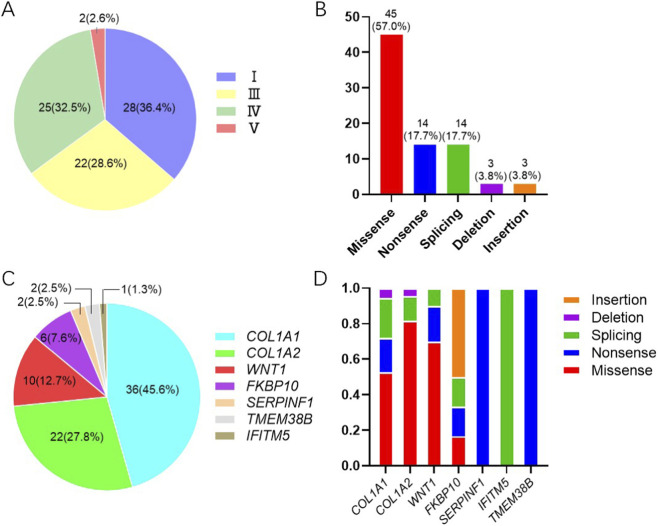
Analyses of clinical types and genetic characteristics in this OI cohort. **(A)** Distribution of patients by clinical types. **(B)** Proportion of variant types. **(C)** Proportion of OI patients by mutated gene. **(D)** Proportion of variant types within each gene.

The cohort of the current study comprised 32 probands with family history (41.4%) and 43 sporadic cases (55.8%); two probands were adopted, making it impossible to determine their family history. Total 77 probands (59 adults and 18 children; 42 males and 35 females) were grouped according to the OI clinical classification: 28 with type I (36.4%), 22 with type III (28.6%), 25 with type IV (32.5%), and 2 with type V (2.6%) (Figure 1A). Notably, no patients with type II OI were included in this cohort, as this form is typically perinatally lethal All probands exhibited typical OI features, including recurrent fractures, short stature, and limb deformities. Among the 77 probands, 77.9% (60/77) experienced mild or moderate severity fractures (≤20 times), while the remaining experienced more frequent fractures (17/77, 22.1%), and 45.5% of patients with type III OI experienced over 20 fractures. Other clinical manifestations included scoliosis (24.6%, 19/77), limited mobility (31.1%, 24/77), ligament laxity (27.3%, 21/77), grey or blue sclerae (53.2%, 41/77), and DI (58.4%, 45/77). Only two probands had hearing loss requiring hearing aids, and four expressed abnormal facial features (e.g., midfacial hypoplasia, inverted triangular face).

Differences in clinical manifestations were observed among patients of different OI types. Type III patients exhibited the highest prevalence of limb deformity (90.9%, 20/22), limited mobility (63.6%, 14/22), DI (77.3%, 17/22) and ligament laxity (45.5%, 10/22), as well as an increased number of fracture and more severe malformations. Adult patients with Type III OI showed the most severe phenotypes and the shortest mean height (119 cm; range: 80–150 cm). In contrast, Type I patients presented the mildest phenotypes, characterized by 100% (28/28) blue sclerae, the lowest prevalence of skeletal complications (e.g., scoliosis: 3.6%, 1/28; limited mobility: 7.1%, 2/28). Compared with adult patients of other OI types, adult type I patients had the tallest mean height (136.4 cm; range: 130–170 cm). Type IV patients displayed moderate phenotypes, with a height range of 110–163 cm, a limb deformity prevalence of 60% (15/25), low rates of blue sclerae (8%, 2/25) and scoliosis (4%, 1/25), and 56% of patients (14/25) with 0–10 fractures.

### 3.2 Genetic characteristics

Genomic analysis was performed in 77 OI families, with an overall diagnostic yield of 100%. A total of 79 pathogenic variants were identified in this cohort, distributed across 7 OI-associated genes. These variants comprised 5 types, including 45 missense, 14 nonsense, 14 splicing, 3 deletion, and 3 insertion (Figure 1B). Specifically, 36 variants (45.57%) were found in *COL1A1* (19 missense, 7 nonsense, 8 splicing, and 2 deletion), 22 (27.85%) in *COL1A2* (18 missense, 3 splicing, and 1 deletion), 10 (12.66%) in *WNT1* (7 missense, 2 nonsense, and 1 splicing), 6 (7.59%) in *FKBP10* (1 missense, 1 nonsense, 1 splicing, and 3 insertion), 2 (2.53%) in *SERPINF1* (2 nonsense), 2 (2.53%) in *TMEM38B* (2 nonsense), and 1 (1.27%) in *IFITM5* (1 splicing) (Figures 1C,D). The recurrent variants included c.1299 + 1G>A and c.3766G>A in *COL1A1*, c.2835 + 1G>A and c.982G>A in *COL1A2*, c.-14C>T in *IFITM5*, and c.507G>A in *TMEM38B*, and each of which was observed in two patients. Additionally, one patient had two different *COL1A1* variants, one patient had two different *COL1A2* variants, and another patient was a double heterozygote of both *COL1A1* and *COL1A2* variants.

In this study, 26.58% (21/79) pathogenic variants were not previously recorded in HGMD. The 21 variants were distributed across 6 genes: *COL1A1* (8 variants), *COL1A2* (5 variants), *FKBP10* (4 variants), *SERPINF1* (2 variants), *WNT1* (1 variant), and *TMEM38B* (1 variant). The novel variants included 9 missense, 5 nonsense, 3 splicing, 2 insertion, and 2 deletion (Table 1). All variants were classified for pathogenicity according to ACMG guidelines (Lindahl et al., 2015).

**TABLE 1:** Twenty-one novel pathogenic variants identified in this study.

Gene	Variant no.	Patient no.	Zygote type	Position	Nucleic acid change	Amino acid change	Variant type	Affected domain	ACMG
COL1A1	1	27	Het	Exon 2	c.253_254insCGCCG	p.Glu85Ter	nonsense	Triple helix	P
	2	5	C-Het	Exon 10	c.719G>A	p.Arg240His	missense	Triple helix	LP
	3	43	Het	Exon 10	c.751-1G>A	p.?	splicing	Triple helix	P
	4	45	Het	Exon 32	c.2128-1G>T	p.?	splicing	Triple helix	P
	5	66	Het	Exon 45	c.3331C>T	p.Arg1111Cys	missense	Triple helix	LP
	6	8	Het	Exon 48	c.3631G>A	p.Asp1211Asn	missense	C-terminal domain	LP
	7	11	Het	Exon 48	c.3712delC	p.Leu1238Ter	nonsense	C-terminal domain	P
	8	63	Het	Exon 48	c.3813_3814insCAGAG	p.Gly1272Ter	nonsense	C-terminal domain	P
COL1A2	9	62	Het	Exon 18	c.911G>A	p.Gly304Asp	missense	Triple helix	P
	10	24	Het	Exon 19	c.1031_1033del	p.Val345del	deletion	Triple helix	LP
	11	19	Het	Exon 30	c.1757G>C	p.Gly586Ala	missense	Triple helix	P
	12	10	Het	Exon 31	c.1819G>A	p.Gly607Arg	missense	Triple helix	P
	13	70	Het	Exon 34	c.2063G>A	p.Gly688Glu	missense	Triple helix	LP
FKBP10	14	46	Hom	Exon 4	c.705_709dupCCTGG	p.Tyr237Trpfs*11	insertion	PPIase domains	P
	15	17	C-Het	Exon 5	c.762_763insCACGTCCTC	p.Phe254delinsPheHisValLeu	insertion	PPIase domains	LP
	16	38	Hom	Exon 5	c.825dupC	p.Gly278Argfs*95	insertion	PPIase domains	P
	17	3	C-Het	Exon 6	c.1016G>A	p.Arg339Gln	missense	PPIase domains	LP
SERPINF1	18	23	Hom	Exon 4	c.559dupA	p.Pro186Ter	nonsense	Serpin domain	P
	19	28	Hom	Exon 4	c.638_639del	p.Asp213Ter	nonsense	Serpin domain	P
WNT1	20	12	C-Het	Exon 2	c.284G>A	p.Trp95Ter	nonsense	Conserved homologous domain of WNT family	P
TMEM38B	21	34	C-Het	Exon 3	c.317C>A	p.Ser106Ter	nonsense	The 4th transmembrane region	P

Het=heterozygous, Hom=homozygous, C-Het=compound heterozygous, P=Pathogenic, LP=Likely Pathogenic.

### 3.3 The correlation between genotypes and phenotypes

We performed a correlation analysis between genotype and phenotype in this study. The *COL1A1* variants most commonly associated with Type I (56.8%, 21/37); the *COL1A2* variants had the highest prevalence of Type IV (45.5%, 10/22); the *IFITM5* only accounted for Type V; variants in autosomal recessive (AR) pathogenic genes primarily corresponded to Type III and Type IV (46.7%, 7/15 each). However, we also found that the variants in an AR gene *TMEM38B* led to Type I in one case.

Correlations between variant types and phenotypes were observed in patients with aberrant proα1(I) and proα2(I). Compared with patients carrying quantitative alterations (n = 9), those with structural defects (n = 30) exhibited more severe clinical phenotypes, including shorter mean height (159.7 cm vs. 128.2 cm), more frequent fractures (>5 fractures: 63.3% vs. 33.3%), and higher rates of severe skeletal deformities such as scoliosis (0% vs. 23.3%) and limited mobility (0% vs. 26.7%). Upon further comparing the structural variants between the α1 (n = 13) and α2 (n = 17) chains, it was discovered that structural variants in the α2 chain are more prone to causing severe manifestations: the lower average adult height (135.2 cm vs. 121.2 cm), higher frequency of extremity deformities (53.8% vs. 70.6%), and more experiences with hearing loss (0% vs. 11.8%). In addition, a certain correlation exists between the position of glycine substitution in α1/2 chains and the severity of the disease: the closer the glycine substitution was to the carboxyl-terminal (C-terminal) of the Triple helix domain, the more severe the clinical phenotype, moreover, substitutions near the C-terminal region (e.g., p. Gly958Asp in *COL1A2* exon 44) were predominantly associated with severe OI phenotypes (e.g., Type III). However, glycine substitutions near the amino-terminal (N-terminal) region of the triple-helical domain (e.g., p.Gly200Val in *COL1A1* exon 8 or p.Gly193Val in *COL1A2* exon 12) were more frequently linked to milder phenotypes (e.g., Type I).

## 4 Discussion

In this study, we recruited 77 Chinese OI families and achieved a 100% detection rate with 21 novel pathogenic variants identified. Our finding expanded the spectra of pathogenic variants and revealed additional novel genotype–phenotype correlations.

The clinical distribution of patients in this study was: 28 Type I (36.4%), 22 Type III (28.6%), 25 Type IV (32.5%), and 2 Type V (2.6%). Compared with a previously reported Chinese cohort, the proportion of Type I patients in this study was significantly higher (36.4% vs. 23.83%), while the proportion of severe Type III patients was obviously lower (28.6% vs. 40.94%) (Zhou et al., 2025). When compared with an international cohort, the proportion of Type I patients was slightly lower (36.4% vs. 43.0%), and the proportion of Type III patients was relatively higher (28.6% vs. 16.8%) (Bardai et al., 2016).

As for the pathogenic variant spectrum, a total of 79 pathogenic variants were identified in this study across 7 OI-related genes. Among these variants, *COL1A1* (45.6%) and *COL1A2* (27.9%) were the most common causative genes, consistent with the overall trends of global OI research (Li et al., 2019; Zhou et al., 2025; Glorieux et al., 2000; Richards et al., 2015; Bardai et al., 2016; Lindahl et al., 2015; Holtz et al., 2023). However, the proportion of *COL1A1*/*COL1A2* variants in this cohort (68.4%) was significantly lower than that reported in both domestic and foreign studies (e.g., 79.6%, 83.6% and 87.8%). Notably, the proportion of AR variants in this study (30.4%) was obviously higher than that in other published cohorts (20.8% and 19.6%) (Li et al., 2019; Zhou et al., 2025). *WNT1* was the most common AR-associated gene (12.7%), accounting for 41.2% of all AR-OI cases—this finding was highly consistent with reports on Chinese AR-OI patients, but significantly different from those in foreign populations, suggesting that *WNT1* was the main pathogenic gene in Chinese OI population (Holtz et al., 2023; Tan et al., 2024). Regarding the novel pathogenic variants, 21 novel variants were found in this study, accounting for 26.6% of all pathogenic variants—a higher proportion than in most existing studies (Li et al., 2019; Zhou et al., 2025; Glorieux et al., 2000; Richards et al., 2015; Bardai et al., 2016; Lindahl et al., 2015; Holtz et al., 2023; Tan et al., 2024).

The above clinical and genetic characteristics of this cohort may be associated with their origin and composition of the patients. Most of our patients were referred from other hospitals and institutions, either because their clinical manifestations of OI were atypical, or because they had already undergone routine genetic sequencing to rule out variants in the candidate genes associated with OI, such as *COL1A1*/*COL1A2*. The unique origins of patients led to distributional differences of clinical classification and pathogenic gene, and resulted in a higher proportion of novel pathogenic variants. The present study further highlighted the unique genetic background and variant spectrum characteristics of Chinese OI population.

Most OI cases are caused by heterozygous variants in the type I collagen-encoding genes (*COL1A1* and *COL1A2*) (Forlino and Marini, 2016). The basic structural unit of type I collagen is the procollagen molecule, a heterotrimer consists of two α1 chains encoded by *COL1A1* and one α2 chain encoded by *COL1A2* (Garibaldi et al., 2022; Lugli et al., 2026). Specifically, α1 chain is composed of the N-terminal domain (1–178 AA), the core triple helix domain (179–1192 AA), and the C-terminal domain (1193–1464 AA); the α2 chain is composed of the N-terminal domain (1–90 AA), the core triple helix domain (91–1104 AA), and the C-terminal domain (1105–1366 AA) (Campanini et al., 2021; Doan et al., 2020). By integrating the clinical and genetic data from our previous cohorts of our team (total 590 *COL1A1*/*COL1A2* variants), we established a more comprehensive correlation between variants in different functional domains of type I procollagen and the severity of OI phenotype. This correlation is thought to be determined by the specific roles of each domain during procollagen synthesis and assembly, with a clear gradient of clinical severity: N-Terminal Domain < core Triple helix Domain < C-Terminal Domain (Symoens et al., 2014; Barnes et al., 2019; Marini et al., 2007). Variants in the N-terminal domain (n = 25) mainly impaired triple helix extension or stability and associated with relatively mild phenotype. Notably, no pathogenic variants in the N-terminal domain of α2 chain were detected in the patients recruited by our team. Although several variants in this region have been documented in the HGMD database, most are associated with mild OI (e.g., Type I) or other connective tissue disorders (e.g., Ehlers-Danlos syndrome), suggesting these variants exert weak pathogenic effects on bone loss and fracture risk, and typically do not cause severe skeletal malformations or meet the clinical diagnostic criteria for OI (Malfait et al., 2013). The majority of variants located within the triple helix domain (n = 527) directly damaged the structural stability and folding efficiency of the triple helix, leading to moderate-to-severe phenotype (Pace et al., 2008). Variants in the C-terminal domain (n = 38) usually caused the most severe, even lethal phenotype, because they severely damaged not only the precise recognition of the α chains but also the initial assembly and modification of triple helix (Marini et al., 2007; Malfait et al., 2013; Pace et al., 2008). Through the above analyses, we found and confirmed the correlation between the variants from the N-terminal to the C-terminal in procollagen and the increasing severity gradient of OI, which provided crucial structural biological evidence for further understanding of the pathogenic mechanism and the better prognosis of OI.

## 5 Conclusion

The present study expands the genetic variant spectrum of OI by identifying 21 novel pathogenic variants. By integrating our current data with previous datasets on large OI cohorts, we further clarified population-specific genetic characteristics and genotype-phenotype correlations associated with collagen functional domains. The variant spectrum of OI established herein not only provides critical evidence for precise diagnosis, genetic counseling, and prenatal diagnosis of OI patients but also lays solid foundation for further investigations into the pathogenic mechanisms underlying OI. In conclusion, our study has important implications for precise medicine and scientific research in the field of OI.

## Data Availability

The datasets presented in this article are not readily available because of ethical and privacy restrictions. Requests to access the datasets should be directed to the corresponding authors.
